# *Mycobacterium leprae* Recombinant Antigen Induces High Expression of Multifunction T Lymphocytes and Is Promising as a Specific Vaccine for Leprosy

**DOI:** 10.3389/fimmu.2018.02920

**Published:** 2018-12-12

**Authors:** Márcio Bezerra-Santos, Marise do Vale-Simon, Aline Silva Barreto, Rodrigo Anselmo Cazzaniga, Daniela Teles de Oliveira, Mônica Rueda Barrios, Alex Ricardo Ferreira, Nanci C. Santos-Bio, Steven G. Reed, Roque Pacheco de Almeida, Cristiane Bani Corrêa, Malcolm S. Duthie, Amélia Ribeiro de Jesus

**Affiliations:** ^1^Laboratory of Immunology and Molecular Biology, Federal University of Sergipe, Aracaju, Brazil; ^2^Department of Health Education, Federal University of Sergipe, Lagarto, Brazil; ^3^Infectious Disease Research Institute, Seattle, WA, United States; ^4^Instituto de Investigação em Imunologia, INCT—CNPq, São Paulo, Brazil; ^5^Department of Medicine, Federal University of Sergipe, Aracaju, Brazil

**Keywords:** leprosy, *Mycobacterium leprae*, ML2028, Multifunctional T cells, immunopathogenesis

## Abstract

Leprosy is a chronic disease caused by *M. leprae* infection that can cause severe neurological complications and physical disabilities. A leprosy-specific vaccine would be an important component within control programs but is still lacking. Given that multifunctional CD4 T cells [i.e., those capable of simultaneously secreting combinations of interferon (IFN)-γ, interleukin (IL)-2, and tumor necrosis factor (TNF)] have now been implicated in the protective response to several infections, we tested the hypothesis if a recombinant *M. leprae* antigen-specific multifunctional T cells differed between leprosy patients and their healthy contacts. We used whole blood assays and peripheral blood mononuclear cells to characterize the antigen-specific T cell responses of 39 paucibacillary (PB) and 17 multibacillary (MB) leprosy patients and 31 healthy household contacts (HHC). Cells were incubated with either crude mycobacterial extracts (*M. leprae* cell sonicate–MLCS) and purified protein derivative (PPD) or recombinant ML2028 protein, the homolog of *M. tuberculosis* Ag85B. Multiplex assay revealed antigen-specific production of IFN-γ and IL-2 from cells of HHC and PB, confirming a Th1 bias within these individuals. Multiparameter flow cytometry then revealed that the population of multifunctional ML2028-specific T cells observed in HHC was larger than that observed in PB patients. Taken together, our data suggest that these multifunctional antigen-specific T cells provide a more effective response against *M. leprae* infection that prevents the development of leprosy. These data further our understanding of *M. leprae* infection/leprosy and are instructive for vaccine development.

## Introduction

Leprosy, or Hansen's disease, is a chronic infectious disease caused by *Mycobacterium leprae* (1). The widespread use of multidrug therapy (MDT) over the last three decades has caused a large reduction in the prevalence of leprosy and, by the threshold of new case incidence of < 1 case per 10,000 individuals suggested by the World Health Organization, has led to elimination as a public health concern in most countries (1–4). Despite these efforts, however, new case detection rates have stabilized over the last few years, and leprosy remains a concern in a number of localized regions in countries such as India and Brazil (5–7).

There is now strong associative evidence that the genetic background and immunological response of the infected individual influence both susceptibility to *M. leprae* infection and outcome of leprosy (2, 4, 8, 9). Antigen-specific T helper 1 (Th1) and T helper 17 (Th17) cells are observed in paucibacillary (PB) patients and are associated with control of *M. leprae* replication (2, 8–16). In contrast, T helper 2 (Th2) and T regulatory (Treg) cells are associated with the multibacillary (MB) presentations that are characterized by heavily infected macrophages and multiple skin lesions (14, 15, 17). While it is well-documented that Th1 cells are strongly associated with protection against *M. leprae* infection, assessment of the T cell response by measuring only IFN-γ production may not fully reflect the protective potential of the response (18). Indeed, multiple studies have failed to discriminate healthy but possibly infected individuals (healthy household contacts; HHC) from PB patients on the basis of antigen-specific IFN-γ production (4, 6).

Several attempts have been made to develop vaccines for the prevention of leprosy but bacille Calmette-Guerin (BCG) is the only vaccine currently available (19). Although systematic meta-analyses indicate that BCG vaccination has a protective efficacy that ranges from 20 to 48% within the general population and can achieve up to 80% protection in individuals with prolonged contact with a leprosy case (6, 20), leprosy remains endemic in regions where BCG immunization is standard practice. A new vaccine is highly desirable to maintain control. The sequencing of *M. leprae* genome has facilitated gene synthesis and the design of recombinant fusion proteins for diagnostic and vaccine development purposes (21). Considerable progress has been made and several antigens have been evaluated in clinical situations to enable reverse vaccinology and subsequent testing in preclinical models. Over the last decade, the Infectious Disease Research Institute (IDRI) has enacted a research program to develop new tools to aid in leprosy control efforts (22–25). The identification of new *M. leprae* antigens that are the targeted by the cellular immune response provide promise for both diagnosis and immunoprophylaxis (24–26). Furthermore, understanding how these antigens are recognized is likely the key to generating a protective response.

Multiparameter flow cytometry has allowed the analysis of T-cell effector functions at the single cell level (18, 27) and has revealed that the quality of the CD4 T cell response can dictate the outcome of various conditions. Several studies have demonstrated that CD4 T cells that secrete only IFN-γ have a limited capacity to develop into memory cells (18, 28) but that the proportion of multifunctional Th1 cells (characterized by their simultaneous secretion of multiple cytokines [IFN-γ, IL-2, and TNF-α; (18, 27–29)] positively correlates with protection against various cancers and infectious diseases, including leishmaniasis (29, 30) and tuberculosis (31, 32). For leprosy, individuals exposed to *M. leprae* infection but who have not developed the disease, or the patients with more benign PB presentations of leprosy can be used to evaluate the immune response and its impact on clinical presentation. Thus, we evaluated the immune response to the crude mycobacterial and recombinant ML2028 proteins and the presence of multifunctional T cells expanded *in vitro* by this antigen.

## Material and Methods

### Ethical Considerations

This project adheres to the protocols of the Brazilian Consul for ethics in research (CONEP). The Ethics and Research Committee of the Federal University of Sergipe approved the study (CAAE 0152.0.107.000-07). All subjects or their guardians in case of minors of 18 years-old signed an informed consent form and then responded to an investigative questionnaire to collect demographic and clinical data.

### Study Subjects and Procedures

Study volunteers were attending in the dermatology ambulatory clinic of the University Hospital of Federal University of Sergipe, Aracaju city, Brazil. Leprosy patients were enrolled prior to treatment by conventional multidrug therapy (MDT), in accordance with the Brazilian Ministry of Health and International Leprosy Association (ILA) standards. Each subject was thoroughly examined for the presence of leprosy, leprosy reactions and neurological disabilities. Inclusion criteria were a clinical diagnosis of leprosy with confirmation by histopathology of skin biopsy or by a positive bacilloscopy. Each patient was classified operationally as paucibacillary (PB), when the patient presented with < 5 cutaneous lesions and bacilloscopy exam was negative, or as multibacillary (MB), when the patient presented with more than 5 cutaneous lesions or with major skin infiltration or a positive result upon bacilloscopy exam. Each patient was also defined as having one of the clinical forms described by Ridley-Jopling (33): indeterminate (IL), tuberculoid (TT), borderline (BL) or lepromatous (LL) leprosy. Healthy household contacts (HHC) were subjects living in close and prolonged contact with the leprosy patients and were commonly the patient's spouse. HHC are more exposed to *M. leprae* antigens/infection than the general population, due to the proximity with the index case. Each HHC was provided clinical exam to exclude the possibility that they could also be a patient. Endemic controls were individuals living in the region but with no known interaction with leprosy patients. Individuals were excluded if they presented with diseases that adversely affect immune function, such as HIV, HTLV-I, diabetes and neurological diseases. A total of 87 subjects were included: 39 PB patients, 17 MB patients and 31 HHC. All immunological evaluations were performed before MDT was initiated.

### Antigen Stimulation in Whole Blood Assay (WBA)

Blood was collected into heparinized tubes (10 IU/ml) then seeded in 24 well-plates and incubated with either: 1 μg/ml Purified Protein Derivative (PPD); 10 μg/ml crude antigen from *M. leprae* (MLCS); 10 μg/ml recombinant ML2028 protein. Blood was incubated with 10 μg/ml phytohemagglutinin (PHA) as a positive control or with media alone as a negative control (RPMI 1640: Gibco, Grand Island, New York, USA). Incubations were conducted for 24 h at 37°C, 5% CO_2_, before plasma collection, as previously described (34). Concentrations of IL-2, IFN-γ, IL-10, and IL-17A were determined by Luminex analyses, according to the manufacturer's instructions (Milliplex kit - Human Th17 Magnetic Bead Panel, Panomics, Affymetrix, Fremont, CA).

### Multiparameter Flow Cytometry

**Figure 1:**
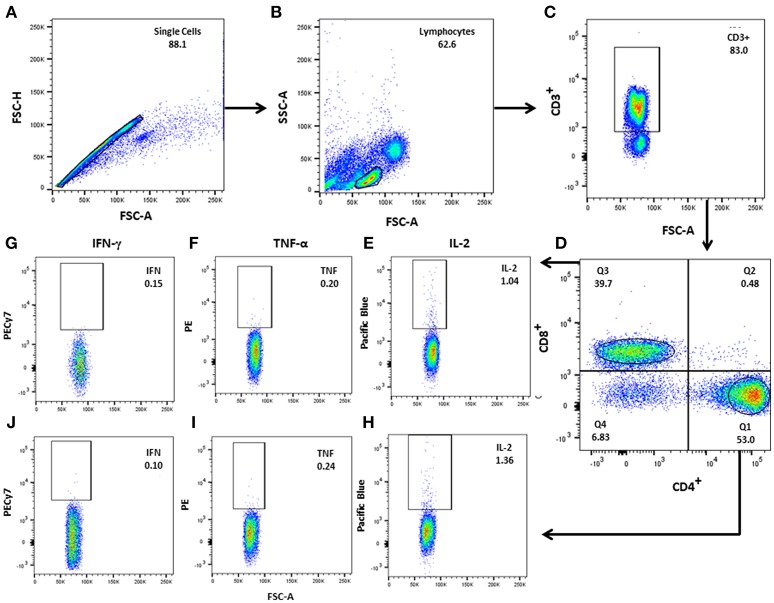
Gating strategy for analysis of multifunctional T cells. **(A)** After single cell selection (FSC-A × FSC-H), lymphocytes were gated according to **(B)** FSC-A (size) vs. SSC-A (granularity), followed by **(C)** CD3^+^ gating. **(D)** CD3^+^CD4^+^ and CD3^+^CD8^+^ T–cell were separated in the CD3^+^ population. **(E–J)** CD3^+^CD4^+^ and CD3^+^CD8^+^ T cells were plotted against each individual cytokine: IL-2, TNF-α, and IFN- γ). Boolean gating was performed to determine the frequencies of each potential combination of cytokine-producing CD4^+^ and CD8^+^ cells using FlowJo software.

To identify and quantify multifunctional T cells, peripheral blood mononuclear cells (PBMC) were isolated using Ficoll-Hypaque (Ficoll-Paque PLUS™, GE-Healthcare, Menio-Park, NJ). PBMC from subsets of TT (*n* = 5), LL (*n* = 6), HHC (*n* = 6), and control (*n* = 6) were seeded at 1 × 10^6^ cells/ml in 48 well-plates (Greiner-CELLSTAR®), incubated with PPD (1 μg/ml), MLCS (10 μg/ml) or ML2028 (10 μg/ml) diluted with RPMI 1640 (Gibco, Grand-Island, New York, USA) for 6 h at 37°C, 5% CO_2_, before the addition of GolgiPlug (BD-Biosciences, Franklin Lakes, NJ) for an additional 12 h. At the end of the culture period, cells were washed then incubated with antibodies against the cell identity markers CD3 (V500), CD4 (FITC), and CD8 (PE-Cy5) and against the cytokines IL-2 (BV421), TNF-α (PE), and IFN-γ (PE-Cy7) (BD Biosciences, San Diego, CA), as previously described (34). Cells were resuspended in staining buffer and events acquired using a BD FACS Canto II. Analysis of the acquired datasets was performed using FlowJo (FlowJo-LLCv10) software. To evaluate multiple parameters, we performed Boolean gating analysis (Figure 1) (15). Comparisons of the frequency, median fluorescence intensity (MFI) and integrated MFI (iMFI) were made on a per group basis. iMFI is the value resulting from the multiplication of the frequency of a cytokine-producing cell subset with the MFI for that cytokine (15). All frequencies, MFI and iMFI values are reported after background subtraction of the frequency or iMFI of the identically gated population of cells from the unstimulated comparator from the same individual.

### Statistical Analysis

Cytokine concentrations were compared across the different subgroups (PB, MB, and HHC) and according to clinical forms (IL, TT, BL, and LL). Mean, median and standard deviation of the groups were calculated. D'Agostino and Pearson tests were applied to analyze if the data exhibited normal distribution. As these data did not follow normal distribution, comparisons of means for a given parameter were made by non-parametric *t*-tests (two-tailed, considering unequal variance of groups), and statistical differences between the groups were determined by Mann-Whitney U tests. Correlation between cytokine levels were performed by Spearman correlation test. All analyses were performed by GraphPad Prism software, version 7, with results considered statistically different when a *p* < 0.05 was assessed.

## Results

### Demographic and Clinical Characteristics of Study Groups

No differences were observed between the age of patients presenting as PB or MB, nor when compared to HHC. The proportion of men presenting with MB (58.8%) was, however, higher than that presenting with PB (28.2%; *p* = 0.02; Table 1). In agreement with operational classification MB patients had a greater number of lesions [mean ± standard deviation (SD) 10.24 ± 4.69] than the PB patients (2.13 ±2.66; *p* < 0.0001). The occurrence of reactional episodes was also significantly higher among MB that PB patients (64.7 vs. 30.77%, *p* = 0.01).

**Table 1:** Demographic and clinical characteristics of PB and MB patients and household contacts (HHC).

**Variables**		**PB (*n* = 39)**	**MB (*n* = 17)**	**HHC (*n* = 31)**	***p*-value**
Age	Range (years)	11–84	10–77	25–79	
	Mean ±*SD*	46.87 ± 17.81	40.59 ± 18.76	48.89 ± 10.81	^*^0.19
Gender (male)	*n* (%)	11 (28.2%)	10 (58.8%)	11 (35.5%)	^**^0.02
Lesion number	Range	^*^0–5	2-20	–	^*^ < 0.0001
	Mean ±*SD*	2.13 ± 2.66	10.24 ± 4.69	–	
Leprosy reaction	n (%)	12 (30.77%)	11 (64.7%)	–	^**^0.01
Physical disability	Degree 1 *n* (%)	19 (48.7%)	08 (47.1%)	–	^**^0.28
	Degree 2 *n* (%)	03 (7.7%)	03 (17.6%)	–	^**^0.26

SD, Standard Deviation;

* Mann-Whitney test;

** *Fisher exact test*.

### Antigen-Specific Secretion of Th1 Cytokines by PB and HHC

**Figure 2:**
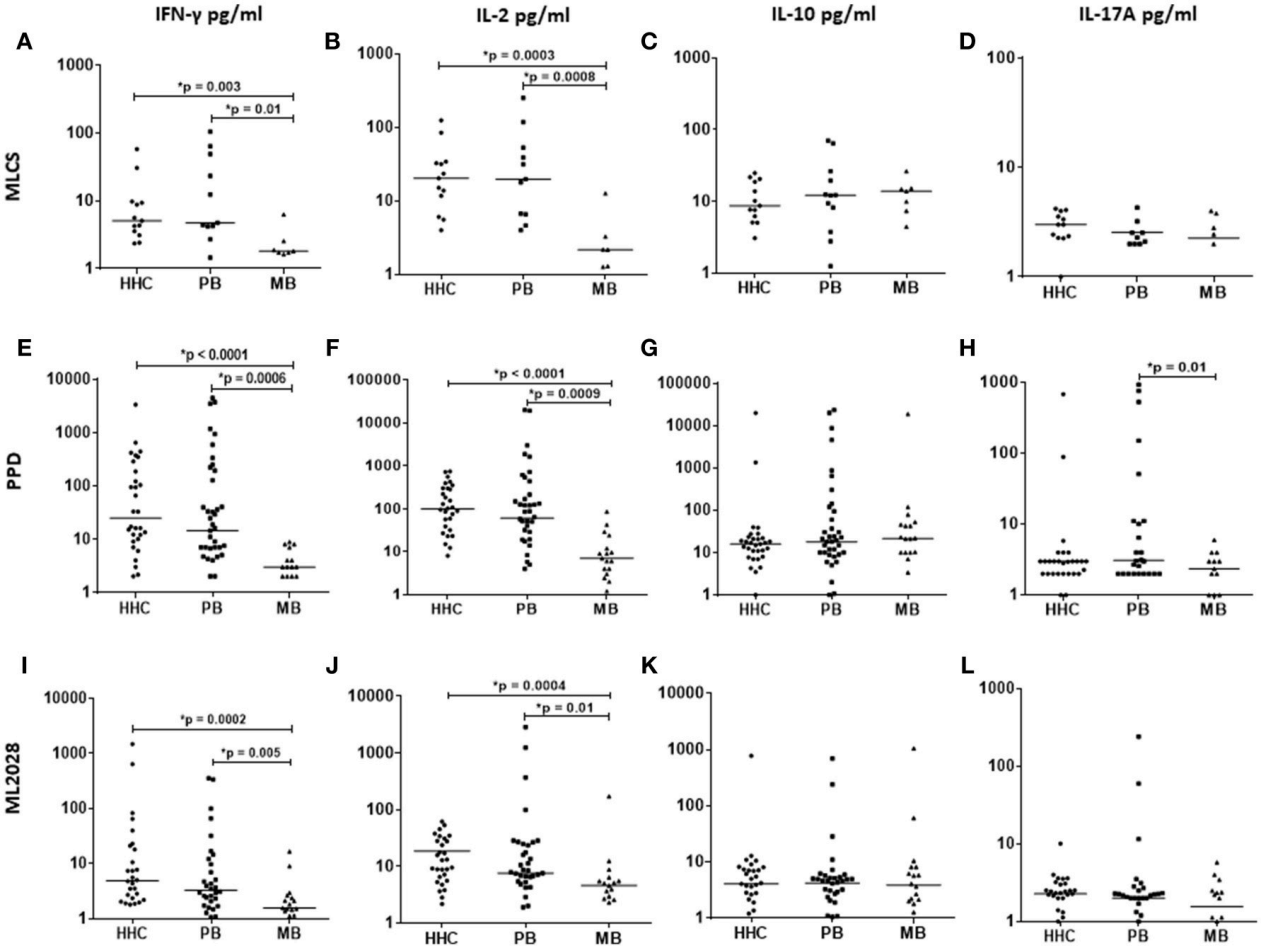
Antigen-specific cytokine production in WBA. IL-2, IFN-γ, IL-10, and IL-17A levels were measured in the plasma collected from WBA incubated with either MLCS (top), PPD (middle), or ML2028 (bottom). Cytokine concentrations were determined by Luminex assay. Each dot represents the results from one recruit, and the horizontal line represents the group mean.

To assess the immune response of recruits, we first measured the cytokines secreted following incubation of whole blood with crude (PPD and MLCS) or recombinant (ML2028) antigens. A recombinant antigen also provides a more robust and reproducible tool for expanded research activities, and ML2028 was selected because it is the *M. leprae* homolog of *Mycobacterium tuberculosis* Rv1886c that encodes the Ag85B protein that has been used extensively in, and well-characterized by, TB research (35). We detected both IFN-γ and IL-2 in WBA of PB patients incubated with PPD and MLCS (Figures 2A–H). Similarly, and suggestive of either exposure to *M. leprae* antigens or even low level infection due to their contact with their index case, whole blood from HHC also secreted IFN-γ and IL-2 following incubation with PPD and MLCS. These results contrasted with those obtained with whole blood from MB patients, where low or undetectable levels of these cytokines were observed (Figures 2A–H). Differences were not observed in IL-10 or IL-17A levels between the groups, both of which were detected only at very low levels. Taken together, these data demonstrate that both PB and HHC possess a Th1-biased anti-mycobacterial response.

We also incubated blood with a recombinant ML2028 protein (Figures 2I–L), observing higher concentrations of IFN-γ in PB (31.5 ± 85.1 pg/ml) and HHC (87.6 ± 298.9 pg/ml) than in MB (2.9 ± 4.05 pg/ml). Similarly, higher concentrations of IL-2 were also observed in WBA with ML2028 in PB (7.59 ± 50.7 pg/ml) and HHC (12.8 ± 16.1 pg/ml) vs. MB (4.6 ± 40.3 pg/ml). As with the crude antigens, IL-10 or IL-17A were not observed in significant levels following incubation with ML2028. These data indicate that responses to ML2028 are similar to those against the whole *M. leprae* bacteria and indicate that ML2028 can therefore be used as a proxy indicator of the anti-*M. leprae* response.

### Multifunctional Antigen-Specific Th1 Cells Are More Abundant in HHC Than Leprosy Patients

**Figure 3:**
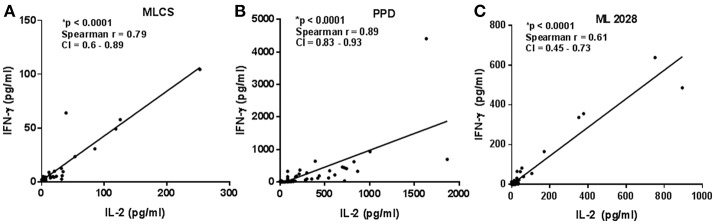
Correlation among supernatant cytokines. Cytokine concentrations from PB (*n* = 23), MB (*n* = 28), and HHC (*n* = 23) samples incubated with either **(A)** ML2028; **(B)** PPD or; **(C)** MLCS were plotted to determine correlation between the IFN-γ and IL-2 by Spearman test. CI, confidence interval. *A indicates highly statistically significant correlations.

The quality of the Th1 response can impact its ability to protect against intracellular pathogens (18), with multifunctional Th1 cells, i.e., those simultaneously producing IL-2, IFN-γ, and TNF-α, appearing preferable to cells secreting only one of these cytokines. We observed positive correlations between IL-2 and IFN-γ concentrations in WBA stimulated with either PPD (Spearman *r* = 0.89, *p* < 0.0001), MLCS (Spearman *r* = 0.79, *p* < 0.0001) or ML2028 (Spearman r = 0.61, *p* < 0.0001) (Figures 3A–C and Supplementary Figure 1) suggesting that these cytokines may be produced simultaneously by multifunctional T cells.

**Figure 4:**
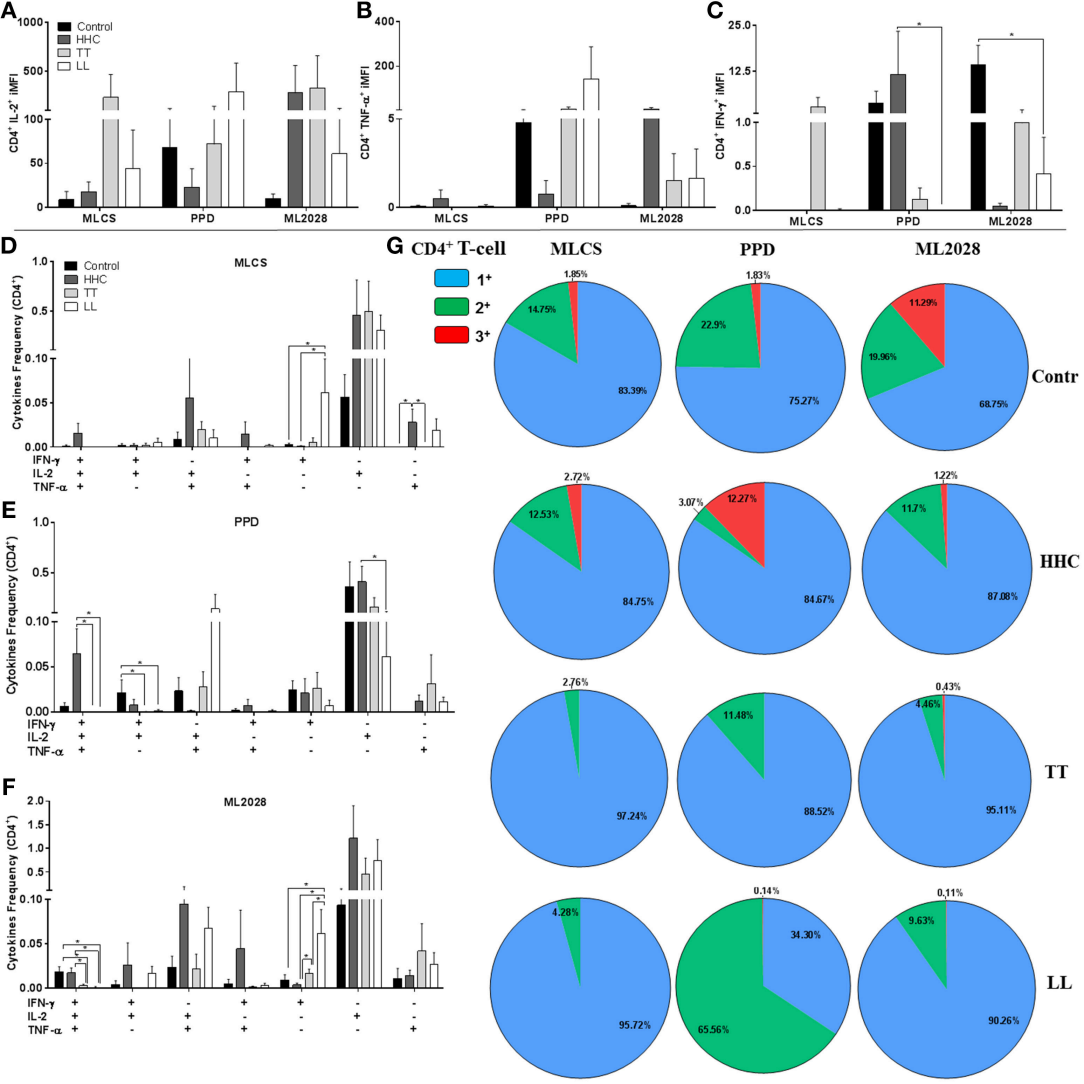
Multifunctional antigen-specific CD4 T cells are more prevalent in HHC than patients. The iMFI of CD4 T cells producing **(A)** IL-2; **(B)** TNF-α or; **(C)** IFN-γ was determined. The frequency of CD4 T cells exhibiting each of possible combinations of cytokines were determined for control, HHC, TT patients and LL patients following incubation with **(D)** MLCS, **(E)** PPD or **(F)** ML2028. In **(G)**, the proportion of antigen-responsive cells producing all three cytokines (3^+^), any two cytokines (2^+^) or any one cytokine (1^+^) are depicted. Statistical analyzes were made by Mann-Whitney and T test, *indicate *p* < 0.05.

To determine the source of IFN-γ and IL-2, we purified PBMC to allow analyses of the phenotype of the antigen-specific cytokine-producing cells by flow cytometry. Differences were not observed in the overall percentage of CD3^+^CD4^+^ lymphocytes in each of the groups (data not shown). To account for both the frequency and the cytokine-producing capacity we assessed the iMFI of IFN-γ^+^, IL-2^+^, and TNF^+^ CD4^+^ T cells after incubation with either MLCS, PPD or ML2028. In agreement with the ELISA data, after incubation with ML2028 flow cytometric analyses revealed high iMFI for CD4^+^IL-2^+^ in samples from HHC (279.6 ± 684.9) and TT patients (329.8 ± 659.6; Figure 4A). We also observed that incubation with PPD revealed high iMFI for CD4^+^TNF-α^+^ in HHC samples (8.7 ± 17.9; Figure 4B), in which iMFI of CD4+IFN-γ+ (11.68 ± 28.6; Figure 4C) was also significantly higher than that measured in LL patients (0.0 ± 0.0). Thus, as expected, the predominant source of antigen-specific IFN-γ and IL-2 in PB patients and HHC were CD4 T cells.

We then used a multi-gating strategy to scrutinize the quality of antigen-specific cells producing these Th1 cytokines. Gates were set to enumerate cells that were producing either all three, or any variant combinations of two, of these cytokines. Analyses of PBMC incubated with MLCS revealed higher proportions of CD4^+^IFN-γ^−^IL-2^+^TNF-α^+^ and CD4^+^IFN-γ^+^IL-2^−^TNF-α^+^ double positive cells in HHC than in TT and LL (Figure 4D). After PPD incubation we also observed larger populations of CD4^+^IFN-γ^+^IL-2^+^TNF-α^+^ in control and HHC groups (Figure 4E). Similarly, incubation of PBMC with ML2028 revealed a larger population of CD4^+^IFN-γ^+^IL-2^+^TNF-α^+^ in control (0.022 ± 0.01) and HHC (0.017 ± 0.01) than in TT (0.003 ± 0.002) and LL (0.001 ± 0.002) (Figure 4F). ML2028 was also recognized by more CD4^+^IFN-γ^+^IL-2^+^TNF-α^−^ (0.026 ± 0.06), CD4^+^IFN-γ^−^IL-2^+^TNF-α^+^ (0.094 ± 0.22), and CD4^+^IFN-γ^+^IL-2^−^TNF-α^+^ (0.044 ± 0.1) cells in samples from HHC. ML2028 incubation, however, revealed relatively more single positive CD4^+^IFN-γ^+^IL-2^−^TNF-α^−^ in LL (0.077 ± 0.05) and TT (0.016 ± 0.01) than in control (0.009 ± 0.01) and HHC (0.003 ± 0.00). When we grouped the percentage of antigen-specific cytokine-producing into triple^+^ (red), double^+^ (green), and single^+^ (blue) T cells (Figure 4G) it was apparent that there was a higher percentage of multifunctional ML2028-specific T cells in control and HHC than in TT or LL patients. Similar observations were made following either MLCS and PPD. No differences in the number of multifunctional T cells were detected if these individuals were household contacts of PB or MB patients. Taken together, these data suggest that multifunctional CD4 T cells that recognize ML2028 recombinant antigen are present in HHC that are highly exposed to *M. leprae* infection but who have not developed disease.

### HHC Possess Multifunctional Antigen-Specific CD8 T Cells

**Figure 5:**
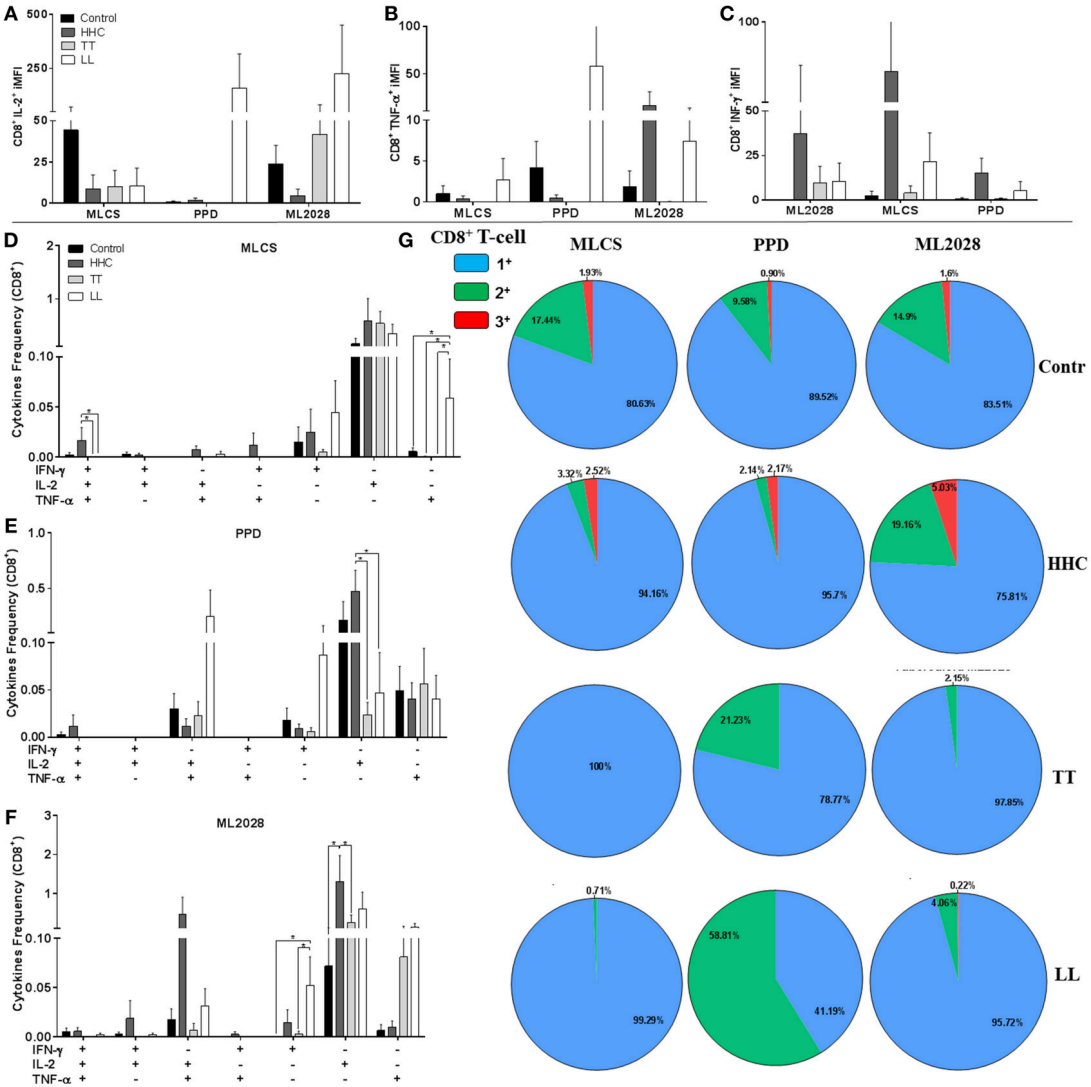
Multifunctional antigen-specific CD8 T cells are more prevalent in HHC than patients. The iMFI of CD8 T cells producing **(A)** IL-2; **(B)** TNF-α or; **(C)** IFN-γ was determined. The frequency of CD4 T cells exhibiting each of possible combinations of cytokines were determined for control, HHC, TT patients and LL patients following incubation with **(D)** MLCS, **(E)** PPD or **(F)** ML2028. In **(G)**, the proportion of antigen-responsive cells producing all three cytokines (3^+^), any two cytokines (2^+^) or any one cytokine (1^+^) are depicted. Statistical analyzes were made by Mann-Whitney and T test, *indicate *p* < 0.05.

Although CD4 T cells are the primary determinant of outcome of *M. leprae* infection/ leprosy, we also assessed the CD8 T cell response. The iMFI of CD8^+^IL-2^+^ in control samples was elevated following incubation with MLCS, but similar values were observed in the other groups (Figure 5A). Cells incubated with PPD had higher CD8^+^IL-2^+^ and CD8^+^TNF-α^+^ iMFI in LL patients (Figures 5A,B), and a small proportion of PPD- and MLCS-specific triple^+^ CD8 T cells were detected in control and HHC (Figures 5D,E, respectively). ML2028 incubation revealed a higher CD8^+^TNF-α^+^ and CD8^+^IFN-γ^+^ iMFI in HHC than that observed for TT and LL patients (Figures 5B,C). In agreement, ML2028 incubation revealed high CD8^+^IFN-γ^+^IL-2^+^TNF-α^+^ expression in control (0.006 ± 0.008) and HHC (0.005 ± 0.009) and a higher percentage of CD8^+^IFN-γ^−^IL-2^+^TNF-α^−^ in HHC (1.55 ± 1.7) than in TT (0.25 ± 0.43) patients (Figure 5F). After grouping positive cells, we observed high proportions of ML2028-specific triple^+^ cells in HHC (5.03%) and control (1.6%; Figure 5G). Incubation with PPD and MLCS, similarly, revealed triple^+^ cells in control and HHC. These data indicate HHC have higher levels of anti-mycobacterial, ML2028-specific multifunctional triple^+^ CD8 T cells than leprosy patients.

## Discussion

Although leprosy presents slowly through the chronic evolution of clinical disease and MDT is generally effective, the current MDT regimen are long and about half of all patients present with reactional episodes during their treatment and some may develop physical disabilities (2, 4, 6, 7). A vaccine is highly desirable as a sustainable strategy for the control the transmission of *M. leprae* and propagation of leprosy. An important protective role has been reported for multifunctional T cells in chronic infectious diseases such as HIV (36), leishmaniasis (29, 30, 37, 38), and tuberculosis (31, 32). It is also indicated that inducing multifunctional antigen-specific T cells leads to the generation of central and effector memory and, such responses are therefore instructive for development of effective vaccines. Despite it being well-established that CD4 T cells are critical determinants of leprosy presentation, this is the first study that has assessed the presence of *M. leprae*–specific multifunctional T cells in leprosy patients and controls. Our data reveal that HHC that do not develop disease have a greater proportion of multifunctional anti-mycobacterial T cells than patients, implicating these cells in protection against the development of leprosy.

A cell-mediated immune response involving both CD4 and CD8 T cells is generally important for controlling infection by intracellular pathogens (18, 37, 38). Moreover, recent studies have also demonstrated that better protection against intracellular infections is provided by vaccines that generate multifunctional CD4 and CD8 T cells (29). According to the cytokines they produce (IFN-γ, IL-2, and TNF-α) multifunctional CD4 T cells, usually have three potential outcomes: they may remain as memory or effector T cells; they may further differentiate into less-functional T cells; or they may die following activation (18). These outcomes can therefore have diverse and important implications for the clinical outcome of infections. We have found that, similar to both PPD and MLCS, incubation with recombinant ML2028 elicited secretion of higher levels of IL-2 and IFN-γ in WBA of HHC and PB samples compared to MB samples. Given that the IFN-γ response could lead to macrophage activation and killing of mycobacteria (2), and that IL-2 could enhance the expansion of Th1 cells (18), these data support previous observation that both HHC and PB can limit *M. leprae* replication and dissemination. These data do not, however, explain how the immune response of HHC is more effective in controlling *M. leprae* infection and prevents disease development.

While BCG has predominantly been used as a vaccine to prevent tuberculosis, it also provides a relative degree of protection against leprosy. Kim and colleagues (39) suggest that BCG contains many antigens that can augment multifunctional T cell populations. In the current study, our data showed also that similar to ML2028, the crude antigen mixtures present in MLCS and PPD were recognized by multifunctional T cells of HHC. Using multiparameter flow cytometry we observed that MLCS, PPD and ML2028 were recognized by larger populations of antigen-specific multifunctional CD4 T cells in control and HHC than in leprosy patients. Given that the CD4^+^IL-2^+^TNF-α^+^ phenotype is associated with central memory and that CD4^+^IFN-γ^+^IL-2^+^ and CD4^+^IFN-γ^+^TNF-α^+^ cells are involved with effector memory generation (18), our data suggest that the larger proportion of multifunctional T cells in HHC could be involved effective control and provides a rational explanation for the resistant to disease development. These data also highlight the importance of using iMFI in determining a total functional response and further suggest that iMFI can be a useful correlate of protection (28). The higher numbers of triple and double cytokine-producing CD4 T cells, and greater iMFI, found in HHC (and even endemic controls) suggests that these multifunctional T cells are at least part of a cell-mediated immune response that prevents progression to clinical disease.

Although focused in multifunctional Th1 cells, our study also sheds light on various other aspects of the anti-*M. leprae* response. The role of CD8 T cells during infection for protection has been also recently investigated (18), with CD8 T cells that produce IFN-γ and TNF-α enhancing cytolytic activity and secreting IL-2 to promote cell expansion that could enhance CD8 T cell memory function (28, 29, 36). While not considered critical for protection against *M. leprae*, our analyses also revealed ML2028-specific multifunctional CD8 T cells in control and HHC. IL-10 (and regulatory T cells) contributes to an equilibrium between inflammatory and anti-inflammatory responses in diseases such as mucosal *Leishmaniasis* and tuberculosis (14, 17, 40–42), but we did not observe IL-10 responses here. We did identify elevated IL-17A levels in WBA with HHC than those using MB samples. A previous study by our group found an association of Th17 response with the milder PB forms of leprosy (16). Taken together, our data indicate a broader and more robust anti-mycobacterial immune response in HHC than that of patients.

In summary, our data demonstrate the presence of multifunctional antigen-specific T cells in both leprosy patients and their contacts who are not diseased. The higher proportions of triple^+^ and double^+^ CD4 and CD8 T cells in HHC, alongside reports from other disease and vaccine studies, implicates these cells in protection against leprosy development. Longitudinal studies contrasting the responses of contacts that develop disease against those that do not are required definitively test this hypothesis.

## Author Contributions

MB-S, RC, DdO, AF, and NS-B acquired data; MB-S, MdV-S, AB, and MB conducted the experiments; MB-S, CC, MD, RdA, and AdJ analyzed data; MB-S, MdV-S, CC, RdA, and AdJ designed research studies; MD, SR, RdA, and AdJ provided material and reagents; MB-S, CC, MD, and AdJ wrote the manuscript. All the authors have read and approved the final manuscript.

### Conflict of Interest Statement

The authors declare that the research was conducted in the absence of any commercial or financial relationships that could be construed as a potential conflict of interest.

## Supplementary Material

Supplementary Figure 1 Correlation among supernatant cytokines. Cytokine concentrations from PB (*n* = 23) and MB (*n* = 28) leprosy patients and HHC (*n* = 23) samples stimulated with MLCS, PPD and ML2028 were analyzed by Luminex Technique and correlation between the cytokines (IFN-γ and IL-2) values were determined by Spearman test. CI, Confidence Interval. *Asterisks indicate statistically significant correlations, at a *p* < 0.05.
