# Supplementary figures and images for: Mycobacterium leprae Recombinant Antigen Induces High Expression of Multifunction T Lymphocytes and Is Promising as a Specific Vaccine for Leprosy
Source: Front Immunol. 2018 Dec 12;9:2920. doi: 10.3389/fimmu.2018.02920 (PMC6315144; doi:10.3389/fimmu.2018.02920)

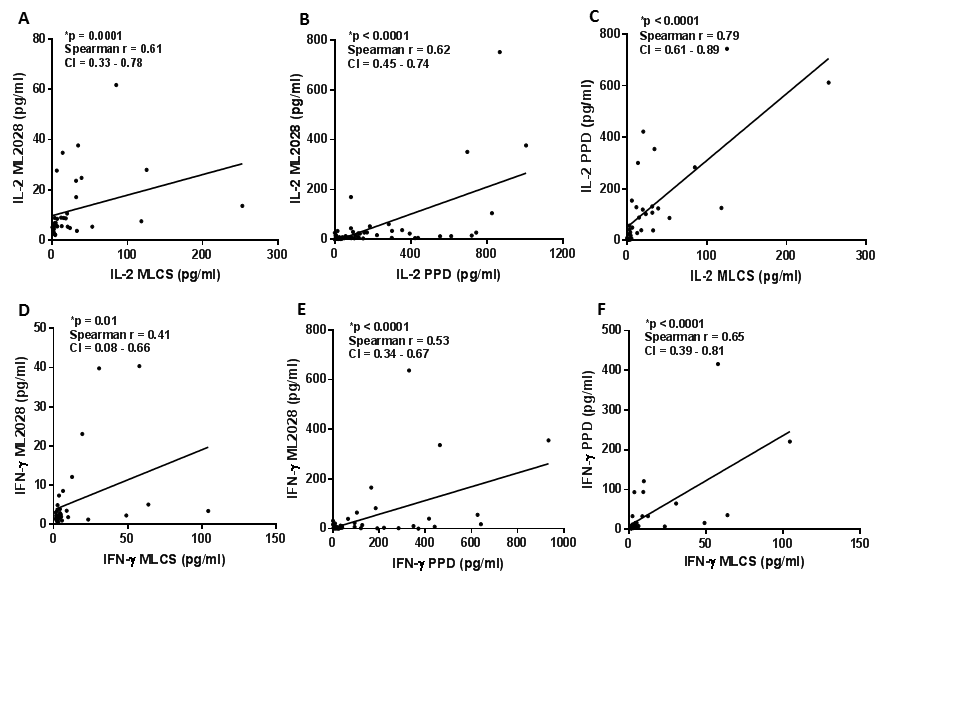

Supplement: Supplementary Figure 1 — Correlation among supernatant cytokines. Cytokine concentrations from PB (n = 23) and MB (n = 28) leprosy patients and HHC (n = 23) samples stimulated with MLCS, PPD and ML2028 were analyzed by Luminex Technique and correlation between the cytokines (IFN-γ and IL-2) values were determined by Spearman test. CI, Confidence Interval. *Asterisks indicate statistically significant correlations, at a p < 0.05. [file Image_1.TIFF]
